# Molecular Profiling of Aggressive Lymphomas

**DOI:** 10.1155/2012/464680

**Published:** 2011-11-24

**Authors:** Maura Rossi, Maria Antonella Laginestra, Anna Gazzola, Maria Rosaria Sapienza, Stefano A. Pileri, Pier Paolo Piccaluga

**Affiliations:** Molecular Pathology Laboratory, Haematopathology Unit, Department of Haematology and Oncology “L. and A. Seràgnoli”, S. Orsola-Malpighi Hospital, University of Bologna, Via Massarenti 9, 40138 Bologna, Italy

## Abstract

In the last years, several studies of molecular profiling of aggressive lymphomas were performed. In particular, it was shown that DLBCL can be distinguished in two different entities according to GEP. Specifically, ABC and GCB subtypes were characterized by having different pathogenetic and clinical features. In addition, it was demonstrated that DLBCLs are distinct from BL. Indeed, the latter is a unique molecular entity. However, relevant pathological differences emerged among the clinical subtypes. More recently, microRNA profiling provided further information concerning BL-DLBCL distinction as well as for their subclassification. In this paper, the authors based on their own experience and the most updated literature review, the main concept on molecular profiling of aggressive lymphomas.

## 1. Introduction

Burkitt lymphoma (BL) and diffuse large B-cell lymphoma (DLBCL) are the commonest aggressive B-NHL worldwide and represent distinct entities in the World Health Organization (WHO) classification [1, 2].

BL is listed in the WHO classification as a single genetic and morphologic entity with variable clinical presentation. It accounts for 30–50% of lymphomas in children, but only 1-2% in adults. In particular, the WHO classification recognizes 3 clinical subsets of BL: endemic (eBL), sporadic (sBL), and immunodeficiency-associated (ID-BL) [1].

The endemic form is the commonest type, being the most frequent childhood cancer in equatorial Africa [1, 3–5]. eBL is almost invariably associated with Epstein-Barr virus (EBV) infection, although local environmental toxics (i.e., *Euphorbia tirucalli*) and coinfection with arbovirus or malaria also appear to be important for its pathogenesis [6–8].

sBL is the most commonly recorded form in the USA and Europe. Contrary to eBL, only ~20% of cases are correlated to EBV [9].

Immunodeficiency-associated BL occurs more commonly in patients infected with HIV (HIV-BL). Intriguingly, because HIV-BL can occur in patients with relatively high CD4 counts, immunosuppression *per se* is not sufficient to explain the relatively high prevalence of BL in this setting [10, 11].

The diagnosis of classical BL rests on the presence of a monotonous infiltrate of medium-sized blastic lymphoid cells that show round nuclei with clumped chromatin and multiple, centrally located nucleoli. The tumor cells have a high proliferation rate and intermingled macrophages containing apoptotic debris lead to the morphological aspect of a “starry sky” pattern [1]. Immunophenotypic features of BL include positivity of tumor cells for CD20 and CD10 (and BCL6), negativity for BCL2, and a proliferation fraction measured by Ki-67 immunohistochemistry of nearly 100%. On the basis of morphology, phenotype, and genetics, BL is currently regarded as a germinal-center- (GC-) derived neoplasm [1]. Notably, according to the somatic hypermutation (SH) patterns and the expression of specific EBV-related molecules, in the WHO classification, a different origin for the endemic and sporadic forms has been suggested [1, 12, 13].

The molecular hallmark of BL is a chromosomal rearrangement of MYC, in form of reciprocal translocation, juxtaposing MYC to the immunoglobulin heavy chain (IGH locus [(t(8; 14)(q24; q32)]) or the *κ* or *λ* light chain loci (t(2; 8)(p12; q24) and t(8; 22)(q24; q11), resp.). Different breakpoints on chromosome 14 and different mutation pattern of the 5′-region of MYC have been recorded between eBL and sBL [14]. In addition, although all BLs have similar phenotype and MYC translocation, it has been demonstrated that the 3 subtypes may have different pathogenetic mechanisms and a key role for EBV has been proposed [13]. 

Diffuse large B-cell lymphoma (DLBCL) is the most common form of lymphoma and accounts for 30–40% of all lymphomas in adults [2]. Clinical outcome is extremely various, with 5-year survival rates between 30% and 80%, widely dependent on clinical risk factors and biological heterogeneity [2]. DLBCL is characterized by the proliferation of large neoplastic B cells, with nuclear size equal to or exceeding normal macrophage nuclei or more than twice the size of a normal lymphocyte, that has a diffuse growth pattern and comprises centroblastic, immunoblastic, T-cell/histiocyte-rich and anaplastic morphological variants [2]. Morphological, biological, and clinical studies have subdivided diffuse large B-cells lymphomas into morphological variants, molecular and immunophenotypical subgroups, and distinct disease entities.

Of note, the differential diagnosis between BL and DLBCL is sometimes unclear, as both entities show overlapping morphological, immunophenotypic, and genetic features [15]. This distinction is also often critical for the different management of these two diseases. In fact, relatively low-dose chemotherapy regimens such as cyclophosphamide, doxorubicin, vincristine, and prednisone (CHOP) are typically used to treat DLBCL, but they can be inadequate for BL [16, 17], for which intensive chemotherapy regimens are required [18–23]. Moreover, the characteristic t(8;14) translocation of BL [24–26] also occurs in 5 to 10 percent of cases of DLBCL [27, 28] and chromosomal breakpoints at the *MYC* locus are recurrently associated with non-IG partner loci and complex chromosomal alterations [24–32]. Because DLBCL is more than 20 times as common as BL [29], a lymphoma with a t(8;14) translocation could present a diagnostic problem. Based on the last WHO classification, B-cell lymphomas with features intermediate between DLBCL and BL have been defined [15] as aggressive lymphomas with morphological and genetic features of both DLBCL and BL, but for biological and clinical reasons, should not be included in these categories. Some of these cases were previously classified as Burkitt-like lymphoma. Most of these cases have intermediate morphological features between DLBCL and BL, with some cells that are smaller than typical DLBCL, resembling BL, and some cells that are larger than typical BL, resembling DLBCL, with a high proliferation fraction, starry-sky pattern, and an immunophenotype consistent with BL. Some cases may be morphologically more typical of BL but have an atypical immunophenotype or genetic features that preclude a diagnosis of BL. The diagnosis of this type of unclassifiable B-cell lymphoma category should not be made in cases of morphologically typical DLBCL that have a *MYC* rearrangement or in otherwise typical BL in which a *MYC* rearrangement cannot be demonstrated. This is a heterogeneous category that is not yet clearly considered a distinct disease entity, but is useful in allowing the classification of cases not meeting criteria for classical BL or DLBCL [15, 30].

## 2. Gene Expression of DLBCL and BL

Even with the use of current diagnostic criteria, the distinction between DLBCL and BL is not always precise; for this reason, in the last years, different molecular studies were performed to further characterize DLBCL and BL.

The first gene expression profiling (GEP) studies revealed that DLBCL is composed of at least two molecularly and clinically distinct diseases [30, 32, 33]. The first subgroup of DLBCL, called germinal center B-cell-like (GCB) DLBCL, expresses genes that are characteristic of normal germinal center B cells and is characterized by frequent REL amplifications, BCL2 translocations, and ongoing somatic hypermutation of the immunoglobulin genes [30, 34, 35]. The second DLBCL subgroup, called activated B-cell-like (ABC) DLBCL, misses expression of germinal center B-cell-restricted genes and resembles mitogenically activated blood B cells. GEP allowed to identify individual genes that predict overall survival in DLBCL, the majority coming from gene expression signatures that reflect the cell of origin, proliferation rate, and host immune response to the tumor. In another couple of studies, a third subgroup of DLBCL has been defined by GEP, termed primary mediastinal B-cell lymphoma (PMBL) [31, 36]. ABC-DLBCL and PMBCL have a constitutive activation of the nuclear factor KB (*NFKB*) pathway that they require for survival, which is not a feature of GCB-DLBCL [31, 36, 37].

These three DLBCL subgroups should be possibly considered different entities since they correspond to different B-cells stages of differentiation, use different oncogenic pathways, and have distinct clinical behaviors [38].

These three subgroups of DLBCL are associated with a different clinical outcome with 5-year survival rates of 59% in GCB-DLBCL, 30% in ABC-DLBCL, and 64% PMBCL [30, 31, 33].

In the last years, two DNA-microarray studies investigated whether global GEP might help to discern BL from DLBCL on the molecular level [39, 40]. In the first [40], 220 mature aggressive B-cell lymphomas that included classical BL, “atypical BL,” and DLBC were studied. A “core group” of eight BLs was defined on the basis of WHO criteria (histological classification of classic or “atypical” BL, CD20+, BCL6+, CD10+, BCL2−, CD5−, KI67 ≥ 95%, and *MYC *translocation). The remaining cases were compared with the BL core group and were labeled with a “BL similarity index” according to gene expression value of 58 genes. On the basis of this index, aggressive B-NHL were classified into molecular Burkitt lymphoma (mBL; 22%), intermediate cases (20%), and nonmolecular Burkitt lymphoma (non-mBL; 58%). The distinctive mBL signature was characterized by 58 genes, including several target genes of the *NFKB* pathway (i.e., *BCL2A1, FLIP, CD44, NFKBIA, BCL3, and STAT3*) that are known to differentiate ABC or GCB lymphomas [30, 32, 33]. These genes were expressed at lower levels in mBL than in cases of GCB DLBCL. This mBL signature was extended also to cases with morphological characteristics of DLBCL and BCL2 positive. Not all cases with a morphologic and immunophenotypical features of BL were classified as mBL. All patients with mBL had a favourable prognosis (5-year survival rate, 75%). Moreover, mBL cases had very few genetic alterations, detected by CGH, in addition to the *MYC* translocation (low genetic complexity); conversely, intermediate and non-mBL cases carried a higher number of chromosomal imbalances (high genetic complexity). Furthermore, non-mBL (DLBCL) cases with the *MYC* translocation had inferior overall survival as compared with DLBCL cases without *MYC* translocation in a retrospective analysis of patients receiving mostly a CHOP/CHOP-like therapy [40].

In the second study [39], 303 cases with the diagnosis of BL, “atypical” BL, and DLBCL were profiled for gene expression. Aggressive B-NHL was first divided into cases in which a MYC target gene signature was identifiable in the total gene expression profile and those in which such a signature was not evident. Expression profiles of the cases that contained a *MYC* target gene signature were compared with the signatures of germinal center B-cell-like (GCB) DLBCL, activated B-cell-like (ABC) DLBCL, and primary mediastinal lymphoma (PMBL). The gene expression-based classifier for BL includes the expression of *MYC* target genes, a particular subgroup of germinal center B-cell-associated genes, and a low expression of major histocompatibility (MHC) class I genes and *NFKB* target genes. Only when all four pairwise comparisons of the gene expression profiles were in favor of the diagnosis of BL, was the diagnosis “molecular BL” assigned. By using this methodology, some cases that were diagnosed as DLBCL or high-grade lymphoma were classified as BL on the basis of gene-expression profiles. This molecular classifier provided a superior quantitative and reproducible diagnosis of BL to the best actual diagnostic method.

Both gene expression studies [39, 40] confirmed that a subset of aggressive B-NHL with evident morphological features of DLBCL showed indeed a gene expression profile of BL. Therefore, both studies help to improve the molecular distinction between BL and DLBCL, but, at the same time, extend the spectrum of molecular BL to some cases that would currently be classified as DLBCL [41]. 

On the basis of GEP data [30, 32, 33], GCB-DLBCL and ABC-DLBCL have a different cellular counterpart and a distinct clinical behavior, as patients with ABC-DLBCL show a poorer outcome than patients with a GCB-DLBCL [30]. Moreover, the molecular classification may be helpful in choosing treatments that can be effective only in specific subtypes of DLBCL. Therefore, GEP can also be an important indication in the management of the patients in the near future. But, GEP analysis is not feasible in routine clinical practice. For this reason, different studies have been made to confirm GEP results using a more easy technical approach such as immunohistochemistry (IHC). IHC can be effected in archival, formalin-fixed, paraffin-embedded tissues and is operable at any pathology laboratory. Different algorithms have been created using the expression of well-known antigens [42–46].

None of the actual immunohistochemical algorithms is able to exactly predict the GEP subtype or to perfectly stratify molecular groups with prognostic value. As a consequence, stratification based on immunohistochemical algorithms for leading therapy is possible only with great caution [47]. 

Importantly, the 2 mentioned GEP studies on BL and DLBCL [39, 40] did not include all BL subtypes. In a most recent paper from our group [48], GEPs of all BL subtypes were studied for the first time and were compared with those of a large panel of B-cell-derived malignancies and normal B lymphocytes. This paper showed that BL was a distinct entity, supporting the current WHO classification [1]. In fact, the GEP of all BL subtypes was quite homogeneous and distinct from those of other lymphomas. Moreover, BL molecular signature could effectively distinguish not only sBL but also eBL and HIV-BL from DLBCL. eBL and HIV-BL shared a common GEPs, whereas sBL cases were relatively more different. The principal differences between eBL and sBL regarded significant pathways such as BCR, TNF/NF-KB, and interleukin-dependent intracellular cascades and probably reflected the different clinical contests. In fact, GEP of eBL, that undergoes a chronic antigen stimulation (e.g., EBV, malaria, and arbovirus infections), had a molecular signature including many genes involved in immune response regulation. Similar considerations could be then applied to HIV-BL. Moreover, a significant difference between eBL and sBL also consisted in the enrichment in genes belonging to the RBL2 network [49, 50]. 

Furthermore, all BL subtypes were definitely related to GC B lymphocytes. In fact, all BL molecular profiles were closer to those of GC lymphocytes than to those of memory cells. However, it cannot be excluded that EBV + BL may origine from a later developmental stage. 

Piccaluga et al. [48] also found a large set of genes differentially expressed in BL and normal GC cells, as a result of malignant event. This set included genes related to immune response, cell cycle regulation, and BCR signalling. These differences may reflect the arrest of differentiation of BL neoplastic cells and the neoplastic transformation [51]. 

 Finally, to further validate gene expression data, immunohistochemistry (IHC) was executed to evaluate the expression of proteins corresponding to genes overexpressed in BL versus normal GC cells and according to potential biologic interest. The 2 molecules CYR61 and SPARC tested by IHC were strongly expressed by the neoplastic cells, suggesting a significant role in BL pathobiology. In fact, CYR61 expression has been related to malignant transformation in different settings, including human lymphomas, being also related to aggressive clinical behavior and drug resistance [52–54], and SPARC contributes to the acquisition of migratory and invasive properties that are recapitulated by malignant tumor cells [42].

## 3. microRNA in BL

Recently, miRNAs have been identified as posttranscriptional regulators of gene expression and involved in physiological and pathological differentiation and maturation processes [55]. They represent a novel mechanism that allows cells to regulate many events such as control cell growth, differentiation, apoptosis, and morphogenesis [56]. Several studies have reported the involvement of miRNAs in cancer [57–59]. 

In particular, in BL, several studies tried to explain some unexplained differences between BL subtypes, for example, *MYC *translocation-positive and *MYC *translocation-negative cases, EBV-positive and EBV-negative cases [60–64]. This is a really important aspect because the *MYC* translocation-negative BL cases may represent a challenging diagnosis to discriminate them from DLBCL and from cases with intermediate features between DLBCL and BL cases (DLBCL/BL) [15, 40, 65, 66].

By using web-available resources (Mirnaviewer, Pic-Tar, Tarbase [67], and miRBase [68]), it was possible to search miRNA directed against a specific target, for example, *MYC*. By using this methodology, Leucci et al. [63] identified hsa-mir-34b. Hsa-mir-34b was found to be downregulated only in BL cases that were negative for *MYC *translocation, suggesting that this event might be responsible for *MYC* deregulation in such cases. Hsa-mir-34b is a member of the mir-34 family, which has been identified as a direct target of p53 and has been observed in human cancers associated with a p53 lack [69]. The hsa-mir-34b downregulation observed in *MYC* translocation-negative cases was not associated with mutations in the hsa-mir-34b gene sequence and probably might be explained with other molecular mechanism. *In vitro* experiments demonstrated that hsa-mir-34b had an impact on MYC regulation. In fact, using a synthetic hsa-mir-34b, a significant dose-dependent decrease of MYC was observed in lymphoblastoid cell lines and using an hsa-mir-34b inhibitor an increase of MYC expression was detected. These findings provided evidence for a novel mechanism of MYC overregulation in BL cases without the *MYC* translocation, as the more common aberrant control exercised by the immunoglobulin enhancer locus. Conversely, the overexpression of hsa-mir-34b in cases with the *MYC* translocation might be due to the loss of regulation on MYC by hsa-mir-34b itself in this condition. In fact, hsa-mir-34b could not be effective in regulating MYC expression, as MYC is under transcriptional control of the immunoglobulin gene promoters. These results are very interesting because they provide a molecular significance of BL *MYC* translocation-negative cases that are often treated as DLBCL. This study showed that MYC upregulation, due to an alternative mechanism, represents a key role in BL pathogenesis also in *MYC* translocation-negative cases.

Interestingly, MYC itself is able to activate the expression of specific miRNAs [55, 56], suggesting the existence of a feedback loop between MYC and specific miRNAs that reciprocally control their expression. MYC overexpression might induce a specific miRNA pattern that, in turn, might be the cause of a differential gene expression and of functional alterations of neoplastic cells [61]. 

In a paper by Onnis et al. [61], a strong upregulation of hsa-miR-17-5p and hsa-miR-20a, which correlates with high levels of MYC expression in BL, was found. Hsa-miR-9* was the only miRNA strongly downregulated only in BL *MYC* translocation-negative cases. 

The hsa-miR-9* expression was tested also in DLBCL and Intermediate DLBCL/BL cases to verify that it is a specific molecular marker for BL cases lacking the typical translocation. 

DLBCL cases showed a strong overexpression of hsa-miR-9*, whereas intermediate DLBCL/BL cases showed a heterogeneous expression, suggesting that the latter subgroup may be considered a heterogeneous category; additional studies are necessary to demonstrate whether hsa-miR-9* may be used as a specific marker to differentiate BL from DLBCL and to identify cases that may benefit from a more aggressive therapy. 

An interesting target of hsa-miR-9* is E2F1, which is essential for the G1-S1 phase passage, which expression is known to be induced by c-MYC, and in turn controls c-MYC expression [70, 71]. 

Interestingly, an inverse correlation between hsa-miR-9* and E2F1 was found. In *in vitro* studies, E2F1 regulation by hsa-miR-9* upregulation reduced E2F1 levels; conversely, miR9* silencing induced E2F1 expression. MYC expression was affected both at the mRNA and protein levels. Thus, hsa-miR-9* inactivation may determine E2F1 upregulation and consequent MYC overexpression in BL lacking *MYC* translocation. The downregulation of hsa-miR-9* in *MYC* translocation-negative cases is caused by an epigenetic mechanism; in fact, an aberrant methylation of hsa-miR-9-1 gene was observed in *MYC* translocation-negative cases. The same epigenetic mechanism was observed in human breast cancer [72]. 

Therefore, this miRNA downregulation may have an important role in the *MYC* negative-translocation BL pathogenesis and may become a molecular signature of these cases, proposing it as a novel candidate for a more careful diagnosis and therapy.

A recent paper by Robertus et al. [64] performed an miRNA expression profile of paediatric t(8; 14) positive and high MYC expressing BL in comparison with *MYC* translocation-negative mantle cell lymphoma (MCL), follicular lymphoma (FL), and chronic lymphocytic leukaemia (CLL). A normal B-cell subset was included to the study. Unsupervised hierarchical clustering analysis showed a unique miRNA profile in BL that was a dominant MYC-induced miRNA profile with most miRNAs being downregulated. The authors observed a significant downregulation for hsa-let7e in BL compared to the three other NHL subtypes. Hsa-miR-150 was also significantly downregulated in BL and targets MYB, which has an essential role in haematopoietic and lymphoid development and apoptosis [73]. Downregulation of hsa-miR-150 in BL compared to the other 3 NHL subtypes might thus result in enhanced MYB levels in BL. The low expression of hsa-miR-26a, that targets EZH2 (a member of the polycomb group of genes) [74], is thus consistent with the previously observed high expression of EZH2 in BL. Also, hsa-miR-155 was found downregulated in BL. Hsa-mir-155 regulates activation-induced cytidine deaminase (AID) expression; in fact, low expression of hsa-mir-155, by the presence of a point mutation in the miR-155 binding site, resulted in increased levels of AID and a higher frequency of MYC translocations [75], consistently with MYC translocation-positive BL. Finally, several other MYC-regulated miRNAs were found to be downregulated in BL, for example, miR-23a/b targeting glutaminase, hsa-miR-125b targeting IRF4 and PRDM1 (BLIMP1), hsa-miR-146a targeting IRAK1 and TRAF6, and hsa-miR-223 targeting LMO2. This study showed a specific miRNA profiling for BL.

Another paper by Leucci et al. [60] showed that hsa-miR-127, which is directed against B-cell regulators, was differentially expressed between EBV-positive and EBV-negative BL cases. In particular, it was highly upregulated only in EBV-positive BL samples, whereas EBV-negative cases showed the same levels of expression with normal controls. Thus, this upregulation may specifically depend on the presence of EBV, though actually the mechanism of hsa-miR-127 regulation by EBV remains unknown. The authors proposed that hsa-miR-127 overexpression in EBV-positive BL may determine downregulation of PRDM1 and XBP-1, and the consequent persistence of BCL6 and germinal center phenotype in a B cell that is already differentiated in terms of surface immunoglobulin and mutation pattern toward a postgerminal center B cells. This further confirms the concept of a different pathogenetic mechanism between EBV-positive and EBV-negative BL. In fact, the upregulation of hsa-miR-127 may be a key event in the pathogenesis of EBV-positive BL, by inhibiting the B-cell differentiation step.

## 4. microRNA in DLBCL

In the last years, several studies were performed to found some differentially expressed miRNAs between DLBCL subtypes.

Lawrie et al. [76] demonstrated that 3 miRNAs were highly expressed in ABC-type but not in GCB-type cell lines; these miRNAs (hsa-miR-155, hsa-miR-21, and hsa-miR-221) were then confirmed to be highly expressed also in *de novo* DLBCL, transformed DLBCL, and follicular lymphoma cases compared to normal B cells. According to the cell line model, microRNAs expression levels were greater in ABC immunophenotype DLBCL cases than in GCB-type immunophenotype DLBCL (*P* < 0.05). Of note, deregulation of miR-155 appears to be a recurrent feature of many malignancies including breast cancer [77], thyroid carcinoma [78], and a range of solid tumors [59]. Moreover, high-grade lymphomas were spontaneously developed by transgenic mice expressing miR-155 targeted to B cells [79]. 

miR-221 has been shown to inhibit normal erythropoiesis through downregulation of c-kit expression [80], but probably this gene is not a significant target for hsa-mir-221 in DLBCL, as no evidence of c-KIT expression was detected by IHC in DLBCL [76].

Overexpression of hsa-miR-21 resulted to be an independent prognostic indicator from IPI status in *de novo* DLBCL, and this high expression was associated with a better prognostic outcome (*P* < 0.05). These 3 microRNAs were investigated also in different developmental stages of B and T cells from healthy individuals; both hsa-miR-155 and hsa-miR-21 were found more highly expressed in ABC than GCB cells. This finding advanced that high levels of these microRNAs in DLBCL are not closely tumor associated but may rather be correlated to a characteristic of their cell of origin [76].

In a paper by Roehle et al. [81], the comparison of miRNA profiles between DLBCL and reactive LN revealed 15 differentially expressed miRNAs. Most differentially expressed miRNAs were downregulated (11 of 15). Hsa-mir-210, hsa-mir-155, and hsa-mir-106A were upregulated; conversely, expression of hsa-mir-149 and hsa-mir-139 was reduced. As previously described, mir-155 seems to play a critical role in B-cell maturation and lymphocyte activation [82, 83]. Moreover, a correlation between mir-155 and NFKB expression was found in DLBCL cell lines and patients [84]. On the other hand, hsa-mir-210 has an important role in cell cycle [85].

A comparison between DLBCL and FL cases showed 10 differentially expressed miRNAs. The expression of hsa-mir-150, hsa-mir-17-5p, hsa-mir-145, hsa-mir-328, hsa-mir-99A, hsa-mir-10A, hsa-mir-95, hsa-mir-151, and hsa-mir-let7e was characteristic of specific DLBCL signature. Hsa-mir-150 was highly downregulated in DLBCL and was found to regulate B-cell development [73, 86]. It has been described that hsa-mir-10A in AML and hsa-mir-17-5p and as hsa-mir-145 were deregulated in various other cancer types [59, 87–90].

In addition, in a paper by Roehle et al. [81], miRNAs with a prognostic relevance were identified. In particular, 8 miRNAs were identified by a multivariate Cox regression analysis including IPI factors. Of these, 6 miRNAs (hsa-mir-19A, hsa-mir-21, hsa-mir-23A, hsa-mir-27A, hsa-mir-34A, and hsa-mir-127) were downregulated and associated with poor EFS and/or OS. Conversely, 2 miRNAs (hsa-mir-195 and hsa-mir-let7g) were correlated to a better prognosis. However, only hsa-mir-127 significantly influenced both OS and EFS in a multivariate analysis and correlated with poor survival prognosis. Hsa-mir-127, reported to be methylated in neoplastic cells and inversely proportional to the expression of BCL6, plays as tumour suppressor [91]. On the contrary, hsa-mir-195 and hsa-mir-let7g, a lower expression of which was associated to a better prognosis, probably function as an oncomiR. Interestingly, in this paper, hsa-mir-155 was not identified as a prognostic marker for survival, although this miRNA was exclusively overexpressed in ABC-DLBCL [76, 92]. 

Malumbres et al. [93] provided experimental evidence that hsa-miR-125b simultaneously downregulated expression of IRF4 and PRDM1 and had an important role in the GC reaction and post-GC differentiation. In fact, hsa-miR-125b expression was higher in GC centroblasts than in memory B cells and was reciprocal to the expression of IRF4 and PRDM1. Moreover, PRDM1 and IRF4 have a complementary binding sites for hsa-miR-125b in the 3′ UTR region and are able to translationally repress expression of both genes through specific interactions with this locus. 

Furthermore, the authors showed that the expression of hsa-miR-223 was low in GC lymphocytes, high in naive and memory B cells, and reciprocal to the expression of LMO2, that is exclusively expressed in GC lymphocytes and has a clinical significance in DLBCL since its expression level is a powerful predictor of survival in DLBCL patients [94, 95]. Noteworthy, hsa-miR-223 was shown to be able to directly regulate LMO2 expression. 

Finally, the authors identified 9 miRNAs whose expression was most different between the DLBCL subtypes; 2 of them (hsa-miR-155 and hsa-miR-21) were previously reported to be differentially expressed between ABC and GCB DLBCL subtypes [76, 96]. Overexpression of hsa-miR-21 was confirmed to be associated with a longer relapse-free survival in a small cohort of 35 not uniformly treated *de novo* in DLBCL cases. The expression level of hsa-miR-21, hsa-miR-155, and hsa-miR-222 was also studied in 106 R-CHOP-treated patients of DLBCL, but only the overexpression of hsa-miR-222, associated with ABC-DLBCL, correlated with shorter OS and PFS. It is unknown if this correlation is caused by a specific function in DLBCL or reflects the ABC subtype [93].

## 5. Conclusion

In conclusion, recent GEP and miRNA profiling studies provided notable information on the molecular pathogenesis of BL and DLBCL. In addition, they provided the basis for a clear distinction of the 2 entities when morphology and phenotype are not conclusive. Finally, the recognition of specific molecular profiles was useful for a more refined subclassification of these lymphomas, with relevant prognostic implication. Certainly, to date, it is difficult to propose global GEP in the routine workup; however, new technologies, including next generation sequencing, will hopefully improve our ability in the molecular managing of lymphoma patients offering adequate surrogates for diagnosis and prognostication.

##  Conflict of Interests

The authors have no conflict of interests to declare.
